# Decentralization and Integration of Advanced Cardiac Care for the World’s Poorest Billion Through the PEN-Plus Strategy for Severe Chronic Non-Communicable Disease

**DOI:** 10.5334/gh.1313

**Published:** 2024-03-27

**Authors:** Sheila L. Klassen, Emmy Okello, Jose M. E. Ferrer, Faraz Alizadeh, Prebo Barango, Pilly Chillo, Yamikani Chimalizeni, Wubaye Walelgne Dagnaw, Jean-Luc Eiselé, Lauren Eberly, Anu Gomanju, Neil Gupta, Bhagawan Koirala, Jacques Kpodonu, Gene Kwan, Bright G. D. Mailosi, Lilian Mbau, Reuben Mutagaywa, Colin Pfaff, Daniel Piñero, Fausto Pinto, Emmanuel Rusingiza, Usman Abiola Sanni, Amy Sanyahumbi, Urmila Shakya, Sanjib Kumar Sharma, Kunjang Sherpa, Isaac Sinabulya, Emily B. Wroe, Gene Bukhman, Ana Mocumbi

**Affiliations:** 1 Center for Integration Science, Division of Global Health Equity, Department of Medicine, Brigham and Women’s Hospital, Boston, United States; 2 Division of Cardiovascular Medicine, Department of Medicine, Brigham and Women’s Hospital, Boston, United States; 3 Department of Medicine, Makerere University, Kampala, Uganda; 4 American Heart Association International, Dallas, United States; 5 Department of Cardiology, Boston Children’s hospital, Boston, United States; 6 Department of Pediatrics, Harvard Medical School, Boston, United States; 7 World Health Organization, Regional Office for Africa, Brazzaville, Republic of Congo; 8 Muhimbili University of Health and Allied Sciences, Department of Internal Medicine, Dar Es Salaam, Tanzania; 9 Kamuzu University of Health Sciences, Queen Elizabeth Central Hospital, Blantyre, Malawi; 10 Center for Integration Science, Division of Global Health Equity, Brigham and Women’s Hospital, Boston, United States; 11 World Heart Federation, Geneva, Switzerland; 12 Division of Cardiovascular Medicine, Department of Medicine, Penn Cardiovascular Outcomes, Quality, and Evaluative Research Center, Cardiovascular Institute, Penn Cardiovascular Center for Health, University of Pennsylvania, Philadelphia, United States; 13 Kathmandu Institute of Child Health, Kathmandu, Nepal; 14 Global Alliance for Rheumatic and Congenital Hearts, Philadelphia, United States; 15 Department of Global Health and Social Medicine, Program in Global NCDs and Social Change, Harvard University, Boston, United States; 16 Department of Cardiothoracic & Vascular Surgery – Manmohan Cardiothoracic Vascular and Transplant Centre, Kathmandu, Nepal; 17 Division of Cardiac Surgery, Department of Surgery, Beth Israel Deaconess Medical Center, Boston, United States; 18 Section of Cardiovascular Medicine, Department of Medicine, Boston University School of Medicine, Boston Medical Center, Boston, United States; 19 Partners In Health, Boston, United States; 20 Department of Global Health and Social Medicine, Harvard University, Boston, United States; 21 Partners In Health/Abwenzi Pa Za Umoyo, Neno, Malawi; 22 Kenya Cardiac Society, Nairobi, Kenya; 23 Department of Internal Medicine, Muhimbili University of Health and Allied Sciences, Dar es Salaam, Tanzania; 24 Jakaya Kikwete Cardiac Institute, Dar es Salaam, Tanzania; 25 Departamento de Ecología Evolutiva, Instituto de Ecología, Universidad Nacional Autónoma de México, Ciudad de México, Mexico; 26 Cardiology Department, Centro Hospitalar Universitário Lisboa Norte, CAML, CCUL, Faculdade de Medicina, Universidade de Lisboa, Lisboa, Portugal; 27 Department of Pediatrics, Pediatric Cardiology Unit, University Teaching Hospital of Kigali, Kigali, Rwanda; 28 College of Medicine and Health Sciences, School of Medicine and Pharmacy, University of Rwanda, Kigali, Rwanda; 29 Partners in Health, Sierra Leone; 30 Department of Paediatrics, Federal Medical Centre, Birnin Kebbi, Nigeria; 31 Pediatric Cardiology, Baylor College of Medicine, Texas Children’s Hospital, Houston, United States; 32 Baylor Center of Excellence, Lilongwe, Malawi; 33 Pediatric Cardiology Department, Shahid Gangalal National Heart Centre, Kathmandu, Nepal; 34 National Academy of Medical Sciences, Kathmandu, Nepal; 35 Cardiology and Internal Medicine, B. P. Koirala Institute of Health Sciences, Dharan, Nepal; 36 Department of Cardiology, National Academy of Medical Sciences, Bir Hospital, Kathmandu, Nepal; 37 Department of Medicine, Makerere University, College of Health Sciences, Kampala, Uganda; 38 Universidade Eduardo Mondlane, Maputo, Mozambique; 39 Instituto Nacional de Saúde, Maputo, Mozambique

**Keywords:** heart failure, advanced cardiac disease, low-income country, decentralization

## Abstract

Rheumatic and congenital heart disease, cardiomyopathies, and hypertensive heart disease are major causes of suffering and death in low- and lower middle-income countries (LLMICs), where the world’s poorest billion people reside. Advanced cardiac care in these counties is still predominantly provided by specialists at urban tertiary centers, and is largely inaccessible to the rural poor. This situation is due to critical shortages in diagnostics, medications, and trained healthcare workers. The Package of Essential NCD Interventions – Plus (PEN-Plus) is an integrated care model for severe chronic noncommunicable diseases (NCDs) that aims to decentralize services and increase access. PEN-Plus strategies are being initiated by a growing number of LLMICs. We describe how PEN-Plus addresses the need for advanced cardiac care and discuss how a global group of cardiac organizations are working through the PEN-Plus Cardiac expert group to promote a shared operational strategy for management of severe cardiac disease in high-poverty settings.

## Introduction

In low- and lower middle-income countries (LLMICs), specialized care for advanced cardiac conditions such as rheumatic and congenital heart disease (RHD and CHD), cardiomyopathies, and hypertensive heart disease (HHD) is delivered predominantly in urban tertiary care settings in urban areas [1]. While concentrating services in urban areas has been the logical first step from a service delivery perspective thus far, the majority of the population in many LLMICs resides in rural areas and receives care mainly at first-level (non-tertiary) referral hospitals as well as health centers [2]. Of the approximately one billion people who live in extreme poverty, half are children and 83% live in rural areas [3]. Cardiac disease in these populations often manifest at late stages as heart failure and, despite a younger mean age at diagnosis, prognosis is much worse compared to other higher-income countries [1,4,5,6]. In this review, we discuss the cardiac aspects of the Package of Essential Noncommunicable Disease Interventions – Plus (PEN-Plus). PEN-Plus is strategy to decentralized advanced cardiac care as part of an integrated management strategy for severe chronic noncommunicable diseases (NCDs). We explore the magnitude of the problem of inadequate cardiac care delivery in LLMICs, as well as the potential of a new global partnerships for advanced cardiac care provision for the world’s poorest.

## Profile of Severe Cardiac Disease in Low- and Lower-Middle Income Countries

Most of what is known about the pattern of advanced cardiac disease in LLMICs comes from urban tertiary referral centers [1]. This data is not necessarily representative of cardiac disease prevalence across the LLMIC population, and population-level incidence and prevalence of advanced cardiac disease in LLMICs is largely unknown. Based on the limited available data, there appear to be major differences in the age distribution of people presenting with cardiac disease, usually manifesting as heart failure in LLMICs as compared with upper-middle-income and high-income countries (HICs) [1,5,7]. The age of presentation for patients with clinical findings of heart failure in low-income countries is younger, with a mean age of 50 years (range: 42–58), compared to the mean age of 70 years (range: 54–77) in upper-middle-income countries. The age at time of heart failure diagnosis correlates with a country’s Human Development Index [1,5]. Further, there is a worse prognosis for those diagnosed with heart failure in LLMICs compared with those in higher income regions [8]. The rate of major cardiovascular events and death is higher among rural compared to urban populations in these settings [7].

HHD is found to be the primary cause of adult heart failure at urban centers in many African countries, followed by RHD, cardiomyopathies, and ischemic heart disease (IHD) [5,9]. In comparison, primary heart failure etiology in India and other low- to mid-income countries in the INTER-CHF study was dominated by IHD, with HHD and dilated cardiomyopathy as other major contributors [9]. Data from rural Rwanda in the largest cohort of heart failure patients from district hospitals in sub-Saharan Africa significantly differs from urban studies. There, primary causes of heart failure were cardiomyopathy, RHD, and HHD among adults, and CHD and RHD among children. Median age in this Rwanda study was 27 years, and 36% of heart failure patients were <18 years old [10].

Pediatric heart failure in LLMICs is less well-studied, but it seems to be primarily attributed to unrepaired CHD and RHD. Unrepaired CHD by itself has been estimated to account for 14% of the noncommunicable disease (NCD) burden in the under 5-year-old age group among the poorest in the world, accounting for a high number of healthy years lost [11]. In a pediatric study done in Malawi, the main congenital causes of cardiac disease were unrepaired ventricular septal defect (24%) and Tetralogy of Fallot (10%), while the major non-congenital diagnosis was RHD (22.4%) [12]. A 7-year registry study of children presenting for care at Uganda Heart Institute showed a prevalence of 27.2% of unrepaired ventricular septal defect and 22% patent ductus arteriosus [13]. In Tanzania, the incidence of critical cardiac conditions at birth was estimated by using pulse oximetry, and hypoxia was found to occur in 2.5 per 1000 live births [14]. This significant burden of CHD, in combination with the lack of cardiac surgical resources for repair, contributes to the high infant mortality in LLMICs.

The initial presentation of patients with cardiac disease is much more severe in LLMICs compared to HICs. Because of limited access to diagnostic services, cardiac disease often presents after clinical heart failure has developed, and often with advanced disease. In rural Rwanda, 40% of admitted patients presented with New York Heart Association class III–IV heart failure [15]. In sub-Saharan Africa (SSA), prognosis for individuals presenting with heart failure is poor; a study in the Democratic Republic of Congo (DRC) reported an in-hospital mortality of 19%, which was significantly associated with poor renal function [16]. One-year mortality after diagnosis of heart failure in SSA has been reported as high as 57.9% in one meta-analysis, probably related to the underlying etiology, access to diagnostics, and availability of evidence-based medical therapy [4].

Traditionally, global policy on cardiovascular disease has been framed around prevention of ischemic heart disease through behavioral modification such as smoking cessation and physical exercise. However, the poorest billion suffers from a large burden of disease that is not cardiometabolic, caused by a diverse set of economic and social factors related to poverty. Given the vastly different demography and causes of cardiac disease in LLMICs, care models used in HICs are insufficient for LLMICs. A reframing is needed in the interest of equity so that strategies can be implemented to prioritize the unique needs of the poorest.

## Access to Advanced Cardiac Care in Low- and Lower-Middle Income Countries

While the large mortality benefit of medications for chronic heart failure with reduced ejection fraction (HFrEF) has been well-established for decades [17], patient access to an evidence-based medication regimen and cardiac diagnostics is limited in many LLMICs. In a recent analysis of service provision assessments, availability of HFrEF medication is variable [18]. Beta-blockers are available in 10% of public first-level hospitals in the Democratic Republic of Congo (DRC), compared to 21% of facilities in Malawi and 69% of facilities in Ethiopia [18]. Angiotensin converting enzyme inhibitors (ACEi) are more available and found in 38% of facilities in the DRC, compared to 49% in Malawi and 80% in Ethiopia [18]. In a survey of 340 facilities in Kenya and Uganda [19], 23% of Kenyan and 22% of Ugandan facilities had availability of all three of furosemide, a beta-blocker, and ACEi. In the same survey, ultrasound machines were available at 38% and 46% of Kenyan and Ugandan hospitals respectively, though researchers were unable to determine whether they were used for cardiac imaging [19].

LLMICs face a critical shortage of healthcare providers. The ratio of physicians to the population in SSA is approximately 1:7000, the bulk of whom are generalists and not specialists [20]. These physicians predominantly live and work in urban areas [21], though some may travel to nearby rural areas periodically to provide care. There are approximately 0.03 pediatric cardiac surgeons per million population in LLMICs, compared to 1.67 per million in HICs [22]. The ratio of nurse to population is slightly better at 1:1200, but this still represents a critical shortage, especially when compared to the nurse to population ratio in North America of 1:120 [20]. There are other providers in LLMICs–such as clinical officers, physician assistants, and nurses with disease-specific training–who perform roles traditionally attributed to nurse practitioners and physicians in HICs. We will be referring to this group of healthcare workers as “mid-level providers”.

Seeking medical care at urban referral centers significantly impoverishes patients living in rural areas. In rural Haiti, most patients needed to borrow money or sell belongings in order to pay for healthcare [23]. The costs for cardiac care are a combination of direct medical costs, transportation costs, and loss of potential income. Distance to a health care facility also has a clinically important impact on care delivery; this is particularly important considering that the majority of the population in LLMICs is rural [2,24]. Even in HICs with a universal healthcare system, urban patients have been found to more likely to receive outpatient care and less likely to require hospitalization within their first year of heart failure diagnosis than their rural counterparts [25]. Compounding these challenges, the INTER-CHF trial found that two-thirds of African patients lacked insurance for healthcare or medications [9].

High quality palliative care, another essential aspect of advanced cardiac care, is nearly absent in LLMICs. Worldwide, death from cardiovascular disease constitutes the highest need for palliative care (38.5% of needs), and the highest proportion of adults and children in need of palliative care at end of life (78% and 98%, respectively) are located in LLMICs [26]. Advanced cardiovascular conditions require holistic care, access to which is relatively unavailable in LLMICs. This unmet need is described in a poignant study using in-depth interviews of advanced heart failure patients in Kenya, in which patients expressed physical, psychosocial, spiritual, and financial distress [27]. Interviews demonstrated that patients did not have adequate information about their illness and that accumulating costs were a barrier to continuity of care and caused tension in social relationships at end of life [27].

## Shifting Modes of Cardiac Care Delivery in Low- and Lower-Middle Income Countries

In HICs, chronic management of heart failure patients is transitioning away from care delivered directly by physicians. The advantages of outpatient, nurse-led, physician-supervised heart function clinics is well-substantiated; the literature reports many advantages including decreased re-hospitalization rates [28,29,30], optimization of an evidence-based medication regimen [29], improved left ventricular ejection fraction [29], and decreased mortality [28,29,31]. Nurse-led management of chronic heart failure patients has also been shown to increase patient satisfaction [32].

There is emerging literature showing that heart failure care delivered outside of tertiary care settings can also be effective outside North America and Europe. Heart failure clinics providing patient and caregiver education have been established in Thailand and demonstrated improved quality of life for patients and sense of control for caregivers [33]. A study in Kuwait demonstrated that training nurses in dedicated heart failure care increased patients’ understanding of their diagnoses [34]. Heart failure care by physicians exists at the referral hospital level in urbanized areas in LLMICs [35]. However, to our knowledge, there are currently only three studies describing non-physician led heart failure care in rural settings in an LLMIC [36,37] in rural Rwanda and rural Malawi [38].

Due to the critical physician shortage in LLMICs, the role of appropriately trained mid-level providers in non-tertiary care settings will help fill a critical gap on the road to universal health coverage. Task-shifting, or targeted training of mid-level providers to provide diagnostic and treatment tasks that would traditionally be performed by physicians in HICs, has occurred successfully in many settings across LLMICs [39]. In rural western Kenya, task-shifting of hypertension management, which is simpler and more algorithmic than cardiac disease, has been found to improve clinical outcomes [40]. Community health workers trained to provide home counseling for chronic heart failure patients were similarly effective when compared to care at a tertiary care hospital [41]. In Rwanda, nurses in chronic care clinics treated patients with guideline-recommended heart failure medications at rates comparable to heart failure clinics in HICs [10]. These studies, as well as many others [42,43], demonstrate that focused training of mid-level providers in NCDs may be effective to improve health outcomes in chronic heart disease in absence of available physician resources.

As a portable diagnostic modality essential to the diagnosis of structural heart disease, focused echocardiography in rural settings can be easily taught as a component of the task-shifting approach. A model of brief, focused training of non-experts to identify RHD has been studied in a variety of countries, all showing good results [36,44,45,46]. Nurses in rural Vietnamese health centers underwent focused echocardiography training and were able to identify major cardiac pathology (such as left ventricular hypertrophy and left-sided valve regurgitation) with a sensitivity, specificity, and accuracy of 75–85% [46]. An integrated web-based and hands-on approach coupled with ongoing remote mentorship yielded good agreement (kappa=0.80) in the hands of non-expert providers who used echocardiography to diagnose HHD, cardiomyopathy, valvular heart disease, right heart failure, and pericardial disease at a remote referral hospital in Northern Uganda [47].

In Uganda, an innovative partnership has been able to create specialized RHD-specific clinics in four rural locations to decentralize cardiac care [48]. Nurses with targeted cardiac training used handheld ultrasound to diagnose structural heart disease and established a program for maternal prenatal RHD screening in these high-risk areas. They have further explored initiatives such as peer counseling, chronic disease education, and a national RHD registry as part of decentralization initiatives. Telemedicine with transmission of echocardiogram images has been shown to be low-cost and feasible in rural Uganda, with significant impact on patient care [49].

## Development of Decentralized Cardiac Care in Low-Income Countries: The PEN-Plus Strategy

There is evidence that those who are poorer live farther from health facilities and are less likely to be able to seek or remain in care [50]. There are multiple reasons for this, including prohibitive cost, lack of public transit infrastructure, loss of income due to time spent traveling, or physical inability to travel long distances. In addition, fear of medical procedures, lack of awareness about cardiac disease progression, and minimal community education on the value of seeking medical care pose additional barriers.

**Figure 1:**
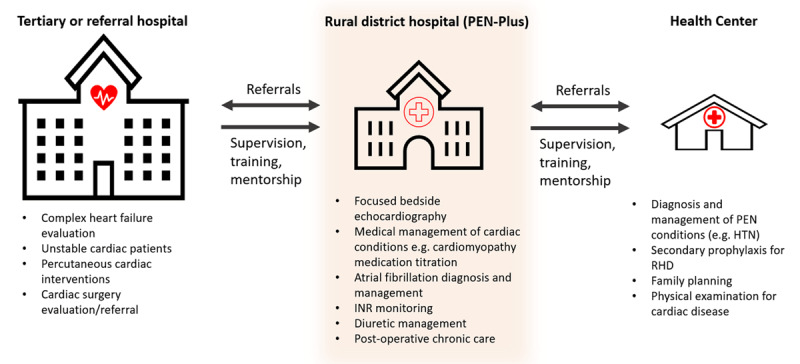
Cardiac service provision at each level of the health system. HTN, hypertension; INR, international normalized ratio; PEN, Package of Essential Noncommunicable Interventions for Primary Care; RHD, rheumatic heart disease.

PEN-Plus is a strategy of integrated care delivery for severe NCDs, acting as a response to the urgent need for decentralization [51]. It complements the World Health Organization (WHO) Package of Essential Noncommunicable Interventions for Primary Care (PEN) adopted in 2010. WHO PEN is a primary care model and set of cost-effective interventions meant to be delivered in low-income settings for common NCDs such as uncomplicated hypertension. As a complement to PEN, PEN-Plus focuses on ambulatory care at the first-referral or district hospital level for severe chronic NCDs such as advanced RHD, CHD, severe hypertension, type 1 diabetes, sickle cell disease, chronic respiratory disease, and chronic kidney disease. PEN-Plus brings care for severe NCDs, including advanced cardiac disease, to the intermediate level of the health system to make care more accessible for the rural poor. It reinforces the facilities’ need for basic diagnostics such as laboratory tests and chest x-rays, as well as a consistent stock of essential medications. PEN-Plus also interacts with primary care at the health center level and advanced care at the tertiary level, resulting in strengthening of the entire health system in which it sits (Figure 1).

PEN-Plus employs task-shifting to address the lack of physician staffing, for example titration of medication dosages for HFrEF by mid-level providers. Key factors enabling task-shifting include targeted training followed by on-site supervision as well as provision of protocols and algorithms for diagnosis and management [42]. Mid-level providers with specialized NCD training have demonstrated the ability to lead outpatient clinics for severe NCDs [36,52], in which delivery of diagnostics such as International Normalized Ratio (INR) testing and focused echocardiography done at the bedside can be practical and effective. Point-of-care INR testing within an NCD clinic helps integrate care for cardiac patients including those with RHD at risk for atrial fibrillation as well as those with mechanical heart valves.

**Figure 2:**
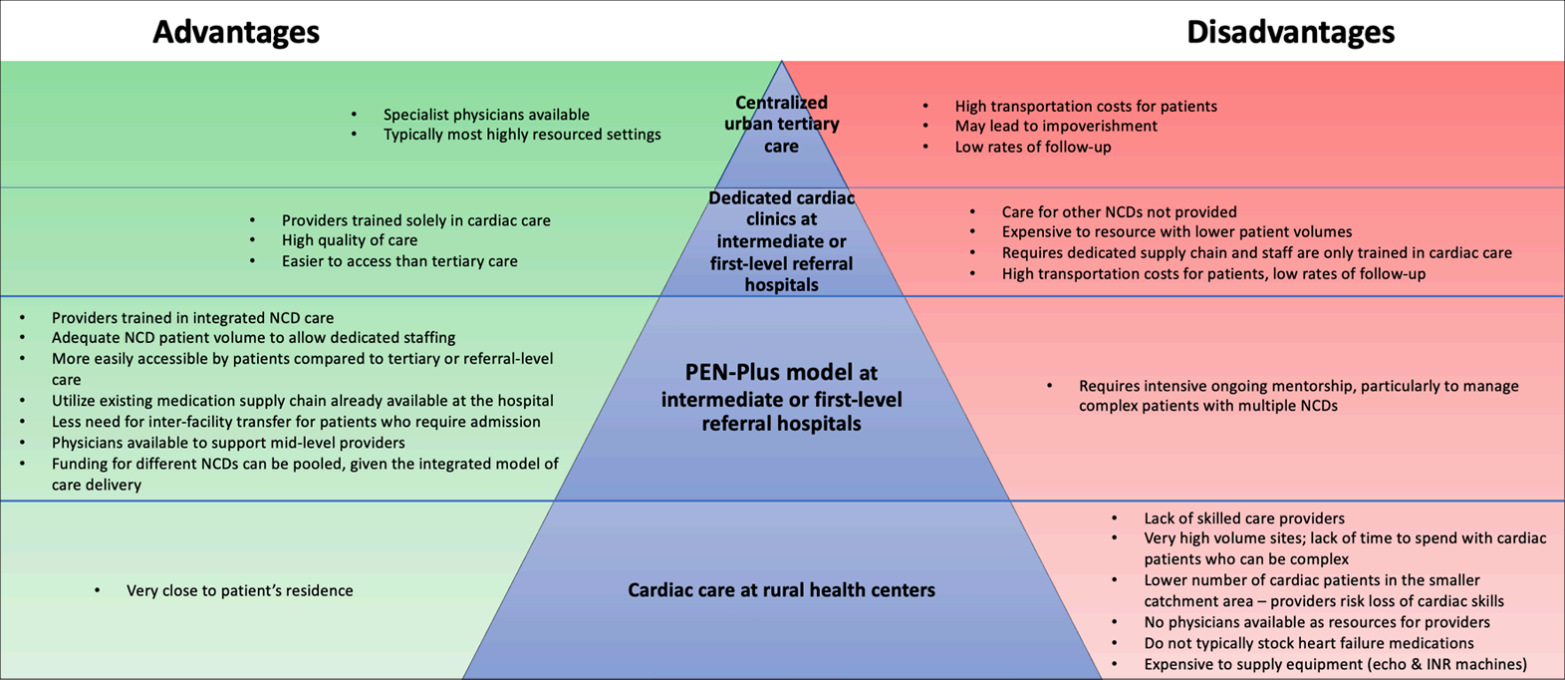
Advantages and disadvantages of cardiac care delivery at different levels of decentralization within the health system. INR, international normalized ratio; NCDs, noncommunicable diseases.

A distinct advantage of PEN-Plus is the ability of providers to care for patients with multiple co-morbidities–many of which are often linked–in an integrated manner, with the intent of preventing re-hospitalization and reducing loss to follow up and mortality. In a Tanzanian study, in-hospital mortality did not differ between heart failure patients versus other inpatients, but the heart failure group had a significantly lower post-discharge one-year survival, highlighting the need for specialized post-discharge cardiac disease follow-up [53]. One notable factor that affects the care delivery model for advanced cardiac care in LLMICs compared to HICs is the volume of patients per center. Establishment of dedicated cardiac clinics is made more challenging in LLMICs due to the low number of healthcare providers facing a large burden of both non-cardiac and cardiac disease. This supports a strategy of having providers trained in management of multiple NCDs in order to provide integrated chronic care. This and other contextual factors require adaptation of decentralization structure in each country setting and a continued integration science approach to care delivery [54]. Figure 2 compares models for delivery of cardiac care in LLMICs at various levels of decentralization.

**Figure 3:**
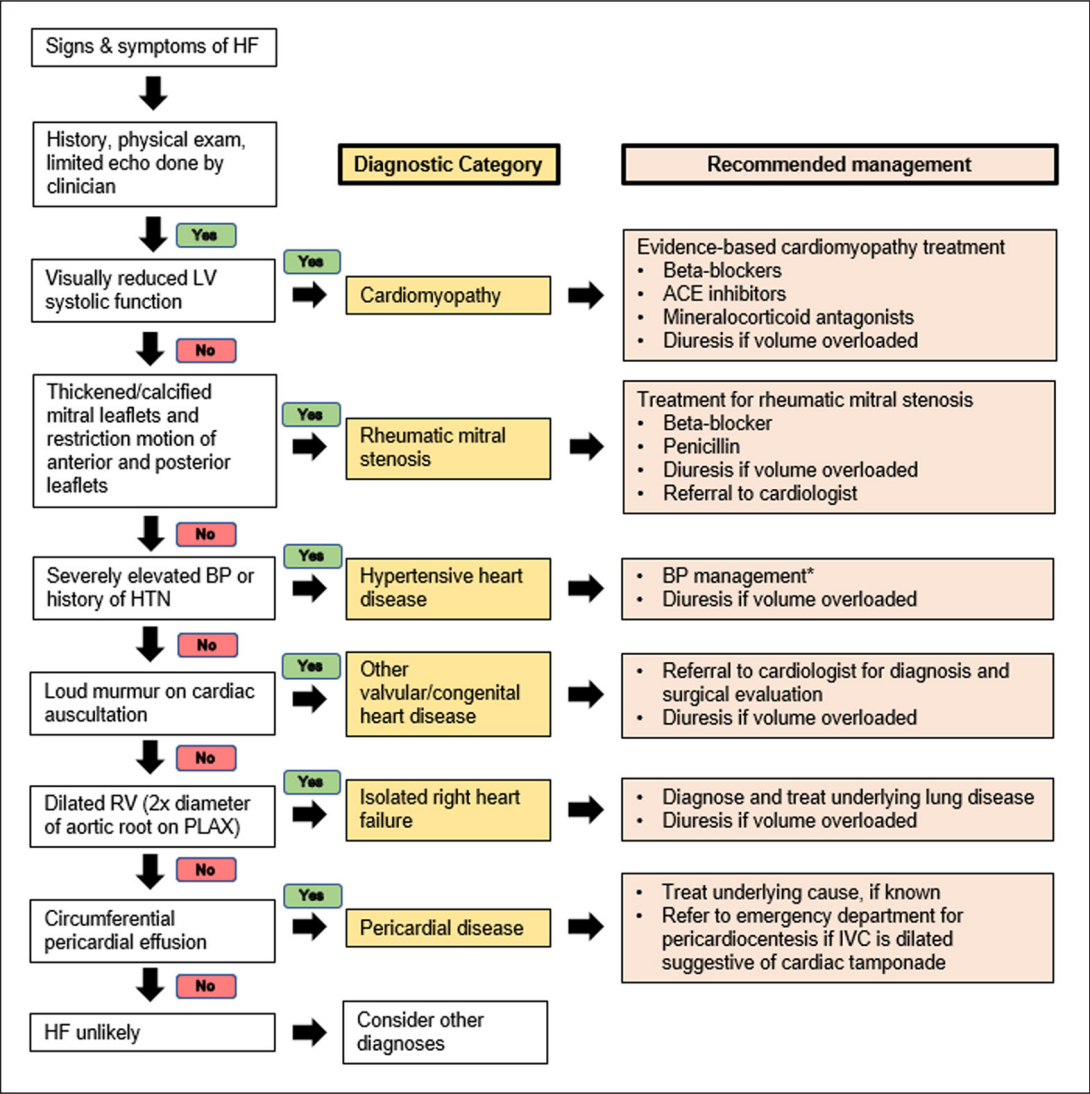
PEN-Plus Heart Failure Management Protocol. ACE, angiotensin converting enzyme; BP, blood pressure; HF, heart failure; HTN, hypertension; IVC, inferior vena cava; LV, left ventricle; PLAX, parasternal long axis; RV, right ventricle *specific medications used will depend on availability on local formularies. This protocol is used primarily for teenagers and older. *Adapted from Bukhman et al*. [51].

The Program in Global Noncommunicable Disease and Social Change at Harvard Medical School began a partnership with Inshuti Mu Buzima (Partners in Health – Rwanda) and the Rwandan Ministry Of Health in 2006 to support nurse-led heart failure clinics as part of a comprehensive PEN-Plus strategy in three rural districts in Rwanda. Nurses learned to perform focused bedside echocardiography using five basic views (parasternal long and short axis, apical four-chamber, subcostal, and inferior vena cava views) and were taught to recognize severe left ventricular systolic dysfunction, mitral stenosis, right ventricular dilation, and pericardial effusion [55]. A simplified management algorithm for teenagers and young adults was developed (Figure 3) based on four years of experience in rural Rwanda, with over 300 patients [56]. Based on this and the published experience of other sites in sub-Saharan Africa at the time of algorithm design [57,58,59], cardiac disease presentations were found to fall predominantly into a limited number of diagnostic categories; findings are usually recognizable on echocardiography or physical examination due to the late presentation of cardiac disease. Categories presented in Figure 3 are meant to be narrow enough to guide specific management, yet broad enough to only require physical examination and focused bedside echocardiography performed by non-physicians for diagnosis. While this approach is not meant to replace a specific cardiac diagnosis made by cardiologists, it can promote timely diagnosis and initiation of appropriate medications. The recommendation for very young children suspected to have cardiac disease is still for direct referral to a pediatrician or pediatric cardiologist due to disease complexity.

When developing this simplified pathway (Figure 3), cardiomyopathy was placed first due to the novice provider’s ability to easily recognize reduced ejection fraction on focused echocardiography and the abundance of evidence that supports a specific medication regimen. Cardiomyopathy is common in rural LLMICs [5,9,10], typically does not require patient transfer outside of the district, and its diagnosis and treatment strengthen diagnostic equipment and medication supply at the intermediate care level. Rheumatic mitral stenosis is next on the protocol because it is endemic in rural LLMIC settings [5,9,10,12], can be difficult to diagnose by physical exam, and has a characteristic echocardiographic appearance at that advanced stage. The specific management pathway for RHD also encourages the strengthening of surgical capacity at the tertiary level in regions where PEN-Plus is implemented. All patients presenting in clinical heart failure with a murmur audible to a mid-level provider should be assessed by a cardiologist once cardiomyopathy, rheumatic mitral stenosis, and HHD have been ruled out. Isolated right heart failure is placed lower on the simplified management pathway since left heart disease, valvular heart disease, and CHD that causes heart failure will eventually cause right ventricular dilation. This algorithm to guide the practice of mid-level providers is not meant to be used in isolation, and ongoing supervision should be provided by local physicians and cardiologists with regular review of patient care [45]. However, this allows for more patients to be managed locally or stabilized prior to transfer to a tertiary care facility.

**Figure 4:**
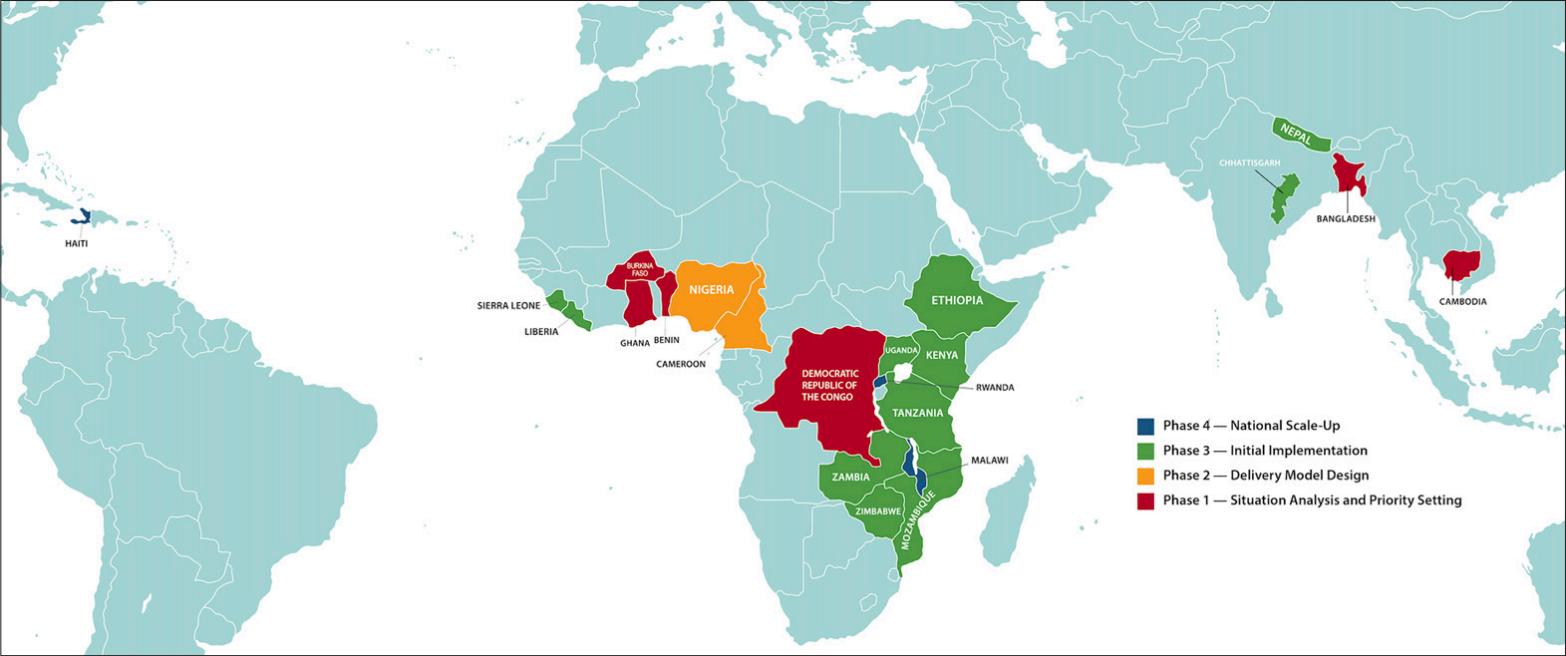
NCDI Poverty Commission Countries and Phases of PEN-Plus Expansion.

Use of this training model in combination with operational support of clinics resulted in a 5-year event-free rate (defined as all-cause mortality, loss to follow-up, and transfer to estimate worst-case mortality) of 41.7% [36]. With training and cumulative experience, nurses were able to decrease five-year heart failure mortality from 38.8% to 27.1% [15], despite environmental and systems challenges. Rwanda is in the Phase 4 (national scale-up) phase of their PEN-Plus implementation (Figure 4).

## Partnering to expand the PEN-Plus strategy: the NCDI Poverty Network

There is the potential to scale PEN-Plus across many LLMICs by aligning stakeholders around a shared operational strategy. In October 2017, leaders from across SSA and Haiti met in Boston, Massachusetts, USA along with representatives from WHO headquarters and WHO Regional Office for Africa (AFRO) to discuss service delivery models for severe NCDs. This was followed by a WHO AFRO Regional Committee meeting in August 2018 during which a PEN-Plus official side event took place, and the PEN-Plus strategy was discussed. A subsequent regional expert consultation in summer 2019 in Kigali, organized by WHO AFRO, was attended by 17 member states, and was centered on discussion of a draft Regional Strategy to support PEN and PEN-Plus expansion in Africa. The PEN-Plus initiative aligns naturally with WHO AFRO’s mission of reducing the NCD burden through collaboration between stakeholders within the health sector and promoting the well-being of all through an integrated management approach [60]. After a four-year process, the Lancet NCDI Poverty Commission published the Lancet Commission on NCDs and Injuries (NCDIs) Report in September 2020, which demonstrated that a diverse set of NCDIs including RHD, CHD, type 1 diabetes, and sickle cell affect impoverished populations early in life and NCDIs comprise the largest gap in achieve universal health coverage for the world’s poorest [61].

These efforts culminated in the formation of the NCDI Poverty Network, launched in December 2020, which is comprised of national NCDI Poverty Commissions as well as technical, policy, and advocacy partners. There are two co-secretariats of the Network: one in Boston, USA, and one in Maputo, Mozambique. The Network provides a platform for global, regional, national, and local leaders to pursue shared research, policy, service delivery, and financing initiatives for severe NCDs. It seeks to elevate an emerging NCDI Poverty movement with the aim of making service delivery for NCDIs a key component to universal health coverage. Figure 4 lists the current NCDI Poverty Network countries at their varying stages of PEN-Plus expansion at the time of writing. One of the strategic initiatives of the NCDI Poverty Network is the PEN-Plus Partnership, which seeks to coordinate collaboration in the areas of advocacy, fundraising, and technical contributions to PEN-Plus implementation and expansion. Organizational participation is facilitated through working groups that meet quarterly and the NCDI Poverty Network High-Level Advisory Group, which includes major partners including UNICEF, the Helmsley Charitable Trust, and the Juvenile Diabetes Research Foundation.

At the 2022 WHO AFRO regional committee meeting in Togo, the 47 member states of the AFRO region voted to adopt PEN-Plus as a Regional Strategy with the goal of having 50% of member states delivering PEN-Plus services at district hospitals by 2025, and 70% by the year 2030 [62,63].

An essential part of the PEN-Plus Partnership is expert consultations with disease-specific stakeholders. The PEN-Plus cardiac consultation took place virtually in February 2021 due to the Coronavirus pandemic, with attendance from numerous major global cardiovascular organizations (Table 1), all of which committed to active support of the PEN-Plus Partnership. These diverse organizations bring years of LLMIC experience in advocacy, public policy, and medical and surgical care for severe cardiac disease to the Partnership. Representatives from all major cardiovascular organizations in attendance voiced the importance of ongoing collaboration within the global cardiovascular community, with policymakers, and with other disease-specific organizations. Four major deliverables were identified for the 2021 to 2023 period: (i) training of cardiologists in NCDI Poverty Commission countries, (ii) development and implementation of e-learning for mid-level providers to accelerate scale-up, (iii) development of a monitoring and evaluation framework, and (iv) execution of a joint strategy for PEN-Plus advocacy. Building on these initial deliverables, goals of the PEN-Plus Partnership to 2030 include supporting PEN-Plus implementation in all interested NCDI Poverty Commission countries and meeting a projected annual financing gap of approximately $100 million. A cardiac expert group was convened in December 2022 with over 40 members from across Phase 3 and Phase 4 NCDI Poverty Network countries (Figure 4). The group consists of two co-chairs representing major organizations within the PEN-Plus Partnership, and includes cardiologists, cardiac surgeons, representatives of cardiac organizations, mid-level providers, and patient advocates from across the NCDI Poverty Network. Major roles of the cardiac expert group are to advise on mid-level provider training and technical materials, discuss and actualize structures for cardiologist support to rural PEN-Plus sites, advise on methods of remote echo mentorship (including the use of telecardiology systems), and brainstorm on novel strategies for PEN-Plus cardiac implementation drawing from experience across multiple low-income countries.

**Table 1:** PEN-Plus Partnership cardiac consultation members*.


American College of Cardiology American Heart Association Cardiac Surgery Intersociety Alliance Chain of Hope Children’s HeartLink Global Alliance for Rheumatic and Congenital Hearts Pan-African Society of Cardiology Resolve to Save Lives Reach World Heart Federation


* Listed alphabetically.

## Cardiovascular Considerations of PEN-Plus Clinic Implementation

Establishing structures to train and support mid-level providers is a major component of initial PEN-Plus implementation. Apart from initial training, which may be done by local or international physicians, structures for ongoing mentorship of clinical service provision and supervised clinical practice must be implemented. If initial training is done by international PEN-Plus faculty, a transition to local supervision must eventually be made. Some Phase 4 PEN-Plus implementation sites (Malawi and Liberia) have been supported virtually by faculty from the Boston NCDI Poverty co-secretariat through a tele-mentoring approach due to the lack of cardiologists in those countries. This has proven effective in improving accuracy of echo interpretation among clinicians [38], but this approach is not sustainable at scale.

A cardiology specialist workforce must be built in PEN-Plus implementation countries to support national scale-up. With mid-level provider adherence to referral guidelines (Figure 3) and establishment of appropriate referral pathways, cardiologists will receive a new cohort of patients from PEN-Plus implementation sites who are suspected to require invasive cardiac interventions for valvular heart disease, need initial diagnosis and management for suspected congenital heart disease, or who are too complex to initially manage at the district hospital level. These cardiologists would be responsible for coordinating percutaneous intervention or cardiac surgery and establishing prioritized procedural waitlists. Some NCDI Poverty Network countries are only supported by a single adult or pediatric cardiologist, and ongoing workforce planning will be discussed by members of the PEN-Plus cardiac expert group. Projection of cardiac disease burden at the district hospital level must be considered as part of workforce planning. Cardiovascular training programs such as the Uganda Heart Institute [64] have already been engaged to train cardiologists for PEN-Plus participation in the African region, where the majority of PEN-Plus implementation countries are located (Figure 4). While most cardiologists in LLMICs will still be based in urban areas, remote supervision can be facilitated by a telecardiology system with digital focused echocardiogram image sharing, and systems for rural cardiology outreach visits can be established.

Approximately 93% of the LLMIC population is estimated to lack access to safe, timely, and affordable cardiac surgical care as a result of workforce, infrastructure, and financial barriers [54]. However, cardiac surgery is an essential service required to prevent morbidity and mortality among patients with valvular and congenital cardiac conditions. PEN-Plus provides the opportunity to identify cardiac conditions earlier in the natural history of the disease, allowing for improved outcomes. As part of PEN-Plus, cardiac surgery and cardiac intervention programs–which will remain highly centralized due to very specialized components such as critical care, perfusion, fluoroscopy, and cardiac-specific anesthesia–need to be developed concurrent to PEN-Plus implementation. Referral for cardiac surgery in the PEN-Plus cardiac framework is through cardiologist consultation (Figure 3) and the use of comprehensive surgical registries; however, counselling and follow-up ultimately occur with PEN-Plus providers. Proper patient selection and prioritization is required due to barriers to surgical access, with an emphasis placed on equity. Social and cultural considerations need to be taken into account when planning for surgery. PEN-Plus clinics are uniquely placed to be able to perform post-operative follow-up of these patients including counselling on family planning, yearly echocardiograms, and, if needed, INR monitoring. Establishment of PEN-Plus clinics are a pre-requisite for the scale-up of local and regional cardiac surgery efforts, since case-finding and post-operative cardiac care are primarily based in PEN-Plus clinic catchment areas. Health systems will need to improve supply chains for cardiac diagnostics and medications as well as strengthen universal health coverage to make care more accessible to all.

## Conclusion

Given the large burden of cardiac disease in LLMICs, poor outcomes after heart failure diagnosis, and significant challenges in access to services among the world’s rural poor, decentralization of cardiac care is essential to bridging the gap in universal health coverage. A reframing of the burden of cardiac disease from cardiometabolic risk factors to social and poverty-related factors emphasizes equity when designing programs for cardiac care delivery in LLMICs. We aim to convey the urgent need to improve cardiac diagnostics, strengthen longitudinal care of individuals with severe cardiac disease, and make cardiac surgery available to the poorest billion to prevent needless death and suffering. Regional and global partnerships are required to implement the PEN-Plus strategy for diagnosis and management of cardiac disease, create local and regional referral pathways for cardiac surgery, and train the cardiac workforce required to support PEN-Plus clinics. Addition of the PEN-Plus strategy into guidelines and policies within Ministries of Health will help integrate severe NCD care into existing healthcare infrastructure. Work of the PEN-Plus cardiac expert group will lead to an improved understanding of cardiac disease in NCDI Poverty Network countries, refinement of structures for cardiac service delivery in rural areas of LLMICs, and advocacy for external financing to support implementation and expansion efforts. Global collaboration with a focus on health system strengthening using innovative strategies is needed to integrate cardiac and severe NCD care for the world’s poorest billion.
