# Correction: Decentralization and Integration of Advanced Cardiac Care for the World’s Poorest Billion Through the PEN-Plus Strategy for Severe Chronic Non-Communicable Disease

**DOI:** 10.5334/gh.1338

**Published:** 2024-06-26

**Authors:** Sheila L. Klassen, Emmy Okello, Jose M. E. Ferrer, Faraz Alizadeh, Prebo Barango, Pilly Chillo, Yamikani Chimalizeni, Wubaye Walelgne Dagnaw, Jean-Luc Eiselé, Lauren Eberly, Anu Gomanju, Neil Gupta, Bhagawan Koirala, Jacques Kpodonu, Gene F. Kwan, Bright G. D. Mailosi, Lilian Mbau, Reuben Mutagaywa, Judith Namuyonga, Colin Pfaff, Daniel Piñero, Fausto Pinto, Emmanuel Rusingiza, Usman Abiola Sanni, Amy Sanyahumbi, Urmila Shakya, Sanjib Kumar Sharma, Kunjang Sherpa, Isaac sinabulya, Emily B. Wroe, Gene Bukhman, Ana Mocumbi

**Affiliations:** 1 Center for Integration Science, Division of Global Health Equity, Department of Medicine, Brigham and Women’s Hospital, Boston, United States; 2 Division of Cardiovascular Medicine, Department of Medicine, Brigham and Women’s Hospital, Boston, United States; 3 Department of Medicine, Makerere University, Kampala, Uganda; 4 American Heart Association International, Dallas, United States; 5 Department of Cardiology, Boston Children’s hospital, Boston, United States; 6 Department of Pediatrics, Harvard Medical School, Boston, United States; 7 World Health Organization, Regional Office for Africa, Brazzaville, Republic of Congo; 8 Muhimbili University of Health and Allied Sciences, Department of Internal Medicine, Dar Es Salaam, Tanzania; 9 Kamuzu University of Health Sciences, Queen Elizabeth Central Hospital, Blantyre, Malawi; 10 Center for Integration Science, Division of Global Health Equity, Brigham and Women’s Hospital, Boston, United States; 11 World Heart Federation, Geneva, Switzerland; 12 Division of Cardiovascular Medicine, Department of Medicine, Penn Cardiovascular Outcomes, Quality, and Evaluative Research Center, Cardiovascular Institute, Penn Cardiovascular Center for Health, University of Pennsylvania, Philadelphia, United States; 13 Kathmandu Institute of Child Health, Kathmandu, Nepal; 14 Global Alliance for Rheumatic and Congenital Hearts, Philadelphia, United States; 15 Department of Global Health and Social Medicine, Program in Global NCDs and Social Change, Harvard University, Boston, United States; 16 Department of Cardiothoracic & Vascular Surgery –Manmohan Cardiothoracic Vascular and Transplant Centre, Kathmandu, Nepal; 17 Division of Cardiac Surgery, Department of Surgery, Beth Israel Deaconess Medical Center, Boston, United States; 18 Section of Cardiovascular Medicine, Department of Medicine, Boston University School of Medicine, Boston Medical Center, Boston, United States; 19 Partners In Health, Boston, United States; 20 Department of Global Health and Social Medicine, Harvard University, Boston, United States; 21 Partners In Health/Abwenzi Pa Za Umoyo, Neno, Malawi; 22 Kenya Cardiac Society, Nairobi, Kenya; 23 Department of Internal Medicine, Muhimbili University of Health and Allied Sciences, Dar es Salaam, Tanzania; 24 Jakaya Kikwete Cardiac Institute, Dar es Salaam, Tanzania; 25 Uganda Heart Institute, Kampala, Uganda; 26 Department of Paediatrics and Child Health, College of Health Sciences, Makerere University, Kampala, Uganda; 27 Departamento de Ecología Evolutiva, Instituto de Ecología, Universidad Nacional Autónoma de México, Ciudad de México, Mexico; 28 Cardiology Department, Centro Hospitalar Universitário Lisboa Norte, CAML, CCUL, Faculdade de Medicina, Universidade de Lisboa, Lisboa, Portugal; 29 Department of Pediatrics, Pediatric Cardiology Unit, University Teaching Hospital of Kigali, Kigali, Rwanda; 30 College of Medicine and Health Sciences, School of Medicine and Pharmacy, University of Rwanda, Kigali, Rwanda; 31 Partners in Health, Sierra Leone; 32 Department of Paediatrics, Federal Medical Centre, Birnin Kebbi, Nigeria; 33 Pediatric Cardiology, Baylor College of Medicine, Texas Children’s Hospital, Houston, United States; 34 Baylor Center of Excellence, Lilongwe, Malawi; 35 Pediatric Cardiology Department, Shahid Gangalal National Heart Centre, Kathmandu; 36 National Academy of Medical Sciences, Kathmandu, Nepal; 37 Cardiology and Internal Medicine, B. P. Koirala Institute of Health Sciences, Dharan, Nepal; 38 Department of Cardiology, National Academy of Medical Sciences, Bir Hospital, Kathmandu, Nepal; 39 Department of Medicine, Makerere University, College of Health Sciences, Kampala, Uganda; 40 Universidade Eduardo Mondlane, Maputo, Mozambique; 41 Instituto Nacional de Saúde, Maputo, Mozambique

**Keywords:** heart failure, advanced cardiac disease, low-income country, decentralization

## Abstract

This article details a correction to: Klassen, S.L., Okello, E., Ferrer, J.M.E., Alizadeh, F., Barango, P., Chillo, P., Chimalizeni, Y., Dagnaw, W.W., Eiselé, J.-L., Eberly, L., Gomanju, A., Gupta, N., Koirala, B., Kpodonu, J., Kwan, G.F., Mailosi, B.G.D., Mbau, L., Mutagaywa, R., Pfaff, C., Piñero, D., Pinto, F., Rusingiza, E., Sanni, U.A., Sanyahumbi, A., Shakya, U., Sharma, S.K., Sherpa, K., Sinabulya, I., Wroe, E.B., Bukhman, G. and Mocumbi, A. (2024) ‘Decentralization and Integration of Advanced Cardiac Care for the World’s Poorest Billion Through the PEN-Plus Strategy for Severe Chronic Non-Communicable Disease’, *Global Heart*. 2024; 19(1): 33. Available at: https://doi.org/10.5334/gh.1313.

## Correction

The following author was not listed by mistake in the original article [1]. This acknowledges that they qualify as one of the co-authors for this paper.

**Judith Namuyonga** 

ORCID 0000-0002-4638-6928

- Uganda Heart Institute, Kampala, Uganda

- Department of Paediatrics and Child Health, College of Health Sciences, Makerere University, Kampala, Uganda

Judith contributed critical content to the paper and also made important edits during the final proofreading process prior to submission.
